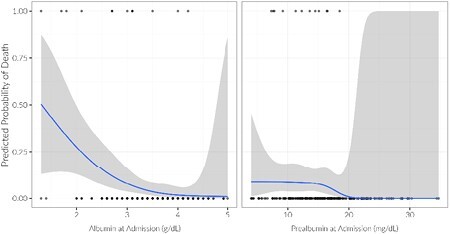# 49 Lower Prealbumin and Albumin Levels in Unhoused Burn Patients Is a Marker for Poorer Outcomes

**DOI:** 10.1093/jbcr/irae036.073

**Published:** 2024-04-17

**Authors:** Sean J Donohue, Noah Speiser, Trevor A Pickering, Christopher H Pham, Justin Gillenwater, Haig A Yenikomshian

**Affiliations:** University of Southern California, Studio City, CA; Keck School of Medicine, University of Southern California, Rolling Hills, CA; University of Southern California, Los Angeles, CA; Keck Medicine of USC, Los Angeles, CA; University of Southern California, Studio City, CA; Keck School of Medicine, University of Southern California, Rolling Hills, CA; University of Southern California, Los Angeles, CA; Keck Medicine of USC, Los Angeles, CA; University of Southern California, Studio City, CA; Keck School of Medicine, University of Southern California, Rolling Hills, CA; University of Southern California, Los Angeles, CA; Keck Medicine of USC, Los Angeles, CA; University of Southern California, Studio City, CA; Keck School of Medicine, University of Southern California, Rolling Hills, CA; University of Southern California, Los Angeles, CA; Keck Medicine of USC, Los Angeles, CA; University of Southern California, Studio City, CA; Keck School of Medicine, University of Southern California, Rolling Hills, CA; University of Southern California, Los Angeles, CA; Keck Medicine of USC, Los Angeles, CA; University of Southern California, Studio City, CA; Keck School of Medicine, University of Southern California, Rolling Hills, CA; University of Southern California, Los Angeles, CA; Keck Medicine of USC, Los Angeles, CA

## Abstract

**Introduction:**

Unhoused (UH) individuals experience burn injuries at a higher rate than the domiciled population, and have poorer outcomes following injuries. One such mechanism proposed for worsened outcomes is secondary to poor nutrition. Access to proper nutrition and food insecurity are major barriers. Malnutrition has been shown to decrease wound tensile strength, increase infection rates, and prolong healing. The purpose of this study was to understand if albumin and prealbumin could help determine outcomes in UH patients and identify at risk patients earlier in their hospital course.

**Methods:**

A retrospective chart review was conducted of UH patients from 2019 through 2023 at a large urban safety net hospital. Data collected included admission laboratory values including albumin and prealbumin. Outcomes studied included length of stay, ICU days, ventilator days, and mortality. Data analysis included Wilcoxon rank sum test and of dichotomous variables using Pearson’s Chi-squared test, zero-truncated negative binomial model for length of stay, a negative binomial hurdle model for ICU length of stay and ventilator days, and logistic regression for mortality.

**Results:**

385 patients met inclusion criteria in the cohort, of which 238 had albumin and 234 had prealbumin information. Each unit (g/dL) decrease in albumin was associated with 1.25 times the length of stay (95% CI = 1.01, 1.55), 1.52 times the odds of being on a ventilator (95% CI = 1.00, 2.29), and 4.46 times the odds of death (95% CI 2.13, 9.98). Each unit (mg/dL) decrease in prealbumin was associated with 1.13 times the odds of death (95% CI 1.02, 1.28). Table 1 demonstrates the relationship of admission albumin across the four patient outcomes studied.

**Conclusions:**

Decreased admission albumin and prealbumin levels are associated with worse burn outcomes in UH patients. These nutritional biomarkers may aid in determining which UH patients are suffering from food insecurity at injury onset. Obtaining these values on admission may help burn providers target nutritional goals in their most vulnerable patients.

**Applicability of Research to Practice:**

Albumin and Prealbumin are associated with worse burn outcomes and thus early nutritional support is of vital importance in the care of UH patients.